# Uncovering a lymphoplasmacytic lymphoma/Waldenström macroglobulinemia initially manifesting as dizziness detected through abnormal serum lipemia index: A case report

**DOI:** 10.1097/MD.0000000000040999

**Published:** 2024-12-20

**Authors:** Kun Wang, Bin Yi

**Affiliations:** a Department of Clinical Laboratory, Xiangya Hospital, Central South University, Changsha, Hunan Province, China.

**Keywords:** biochemical detection, lipemia index, M-proteinemia, Waldenström macroglobulinemia

## Abstract

**Rationale:**

Automated serum index is widely used in biochemical testing, enabling the observation of sample characteristics to a certain extent. The differences between serum lipemia index and sample characteristics can, to a certain extent, facilitate early detection of certain diseases.

**Patient concerns:**

This is a case report of an elderly patient who was admitted to cardiology outpatient clinic due to dizziness.

**Diagnoses:**

Basic tests were normal, but hidden lab tests found abnormal serum index. After discussion, further tests showed high immunoglobulin levels.

**Interventions:**

We performed a lymph node color Doppler ultrasound, blood light chain determination, blood immunization fixed electrophoresis, and urine immunofixation electrophoresis detection. Flow cytometry (lymphoma), FISH (MM + IGH) and MYD88 were detected.

**Outcomes:**

Consequently, the patient was advised to be transferred to the hematology department and was ultimately diagnosed with Lymphoplasmacytic Lymphoma/Waldenström Macroglobulinemia.

**Lessons:**

This case was facilitated by the early detection of discrepancies between lipemia indices and sample appearance despite normal examination results. Additionally, close collaboration between clinical laboratory technicians and clinicians facilitated the uncovering of subtle early disease changes, thereby aiding in precise diagnoses.

## 1. Introduction

M protein is an abnormal immunoglobulin produced in large quantities during the malignant proliferation of plasma cells or B lymphocytes. It is basically an immunoglobulin or immunoglobulin fragment. M-proteinemia is commonly seen in multiple myeloma, malignant lymphoma, macroglobulinemia and other malignant hematological diseases.^[1]^ Immunoglobulin M (IgM) is usually a pentamer formed by 5 Y-type monomers. Owing to its unique physical and chemical properties, this type of M protein can easily precipitate under specific conditions and interfere with colorimetric and turbidity analysis, thus affecting many in vitro test results.^[2–6]^ This leads to inaccurate test results and affects diagnosis and treatment of patients.

In this case report, the patient has lymphoplasmacytic lymphoma/Waldenström macroglobulinemia (LPL/WM), that is usually accompanied by an increase in serum monoclonal IgM gamma globulin.^[7]^ No obvious abnormality was found during the preliminary examination of the patient at the hospital; however, the serum lipemia index was not consistent with the appearance of the sample, therefore, further examination was required to determine this discrepancy. Upon further clarification from the clinic, the doctor suggested that the patient be transferred to the hematology department for early diagnosis. This case is very interesting as the serum lipemia index is used to detect abnormal increase in immunoglobulin. The current findings may improve our in-depth understanding of serum lipemia/turbidity (L), bilirubin/icterus (I), and hemolysis (H) index in the biochemical test.

## 2. Case report

A 70-year-old man was admitted to the cardiology specialist clinic of our hospital due to dizziness. The patient’s laboratory results showed a glycosylated hemoglobin level of 7.20% (reference range 4.20–6.20%) and a uric acid level of 514.8 μmol/L (reference range 208.0–428.0 μmol/L). Routine blood, liver and renal function, fasting blood glucose, blood lipids, myocardial enzymes, and thyroid function were all normal (Table 1), and an electrocardiogram showed no obvious abnormalities. However, the photometric test lipemia index in the lipemia/turbidity, bilirubin/icterus and hemolysis (LIH) of the patient was ++++, yet the specimen serum appearance appeared normal.

**Table 1:** Laboratory results part 1.

Parameter	Results	Reference interval	Units
Hematology			
White blood cells	7.8	3.5–9.5	×10^9^/L
Red blood cells	4.38	4.30–5.80	×10^12^/L
Hemoglobin	130	130–175	g/L
Hct	38.5	40.0–50.0	%
MCV	87.9	82.0–100.0	fL
MCH	29.7	27.0–34.0	pg
MCHC	338.0	316.0–354.0	g/L
Platelets	281	125–350	×10^9^/L
Neutrophils	5.2	1.8–6.3	×10^9^/L
Lymphocytes	1.7	1.1–3.2	×10^9^/L
Monocytes	0.6	0.1–0.6	×10^9^/L
Eosinophils	0.30	0.02–0.52	×10^9^/L
Basophiles	0.04	0.00–0.06	×10^9^/L
Biochemistry tests			
Bilirubin/icterus index	–	–	N/A
Hemoglobin index	–	–	N/A
Lipemia/turbidity index	++++	–	N/A
Total protein	74.6	65.0–85.0	g/L
Albumin	44.4	40.0–55.0	g/L
A/G ratio	1.4	1.2–2.4	N/A
Total bilirubin	15.1	0.0–25.0	μmol/L
Direct bilirubin	5.1	0.0–6.8	μmol/L
Alanine aminotransferase	18.8	9.0–50.0	U/L
Aspartate aminotransferase	18.7	15.0–40.0	U/L
Total bile acid	1.8	0.0–12.0	μmol/L
Urea	4.15	3.60–9.50	mmol/L
Creatinine	72.0	41.0–111.0	μmol/L
Uric acid	514.8	208.0–428.0	μmol/L
Glucose	5.84	5.84	mmol/L
Triglyceride	1.52	<1.70	mmol/L
Cholesterol	3.50	<5.18	mmol/L
High density lipoprotein cholesterol	1.02	1.04–1.55	mmol/L
Low density lipoprotein cholesterol	2.13	1.55–3.19	mmol/L
Lactate dehydrogenase	147.0	120.0–250.0	U/L
Creatine kinase	50.1	50.1–310.0	U/L
Creatine kinase isoenzyme	10.5	<24.0	U/L
Myoglobin	22.6	<70.0	μg/L
Glycosylated hemoglobin	7.20	4.20–6.20	%
Thyroid function			
FT3	5.280	3.1–6.8	pmol/L
FT4	12.750	11–24	pmol/L
TSH	3.330	0.27–4.2	μIU/mL
TgAb	22.510	0–115.00	IU/mL
TRAb	1.70	0–1.75	IU/L

FT3 = free triiodothyronine, FT4 = free thyroxin, Hct = hematocrit, MCH = mean corpuscular hemoglobin, MCHC = mean corpuscular hemoglobin concentration, MCV = mean corpuscular volume, TgAb = antithyroglobulin antibodies, TRAb = thyrotropin receptor antibodies, TSH = thyroid-stimulating hormone.

**Figure 1.**
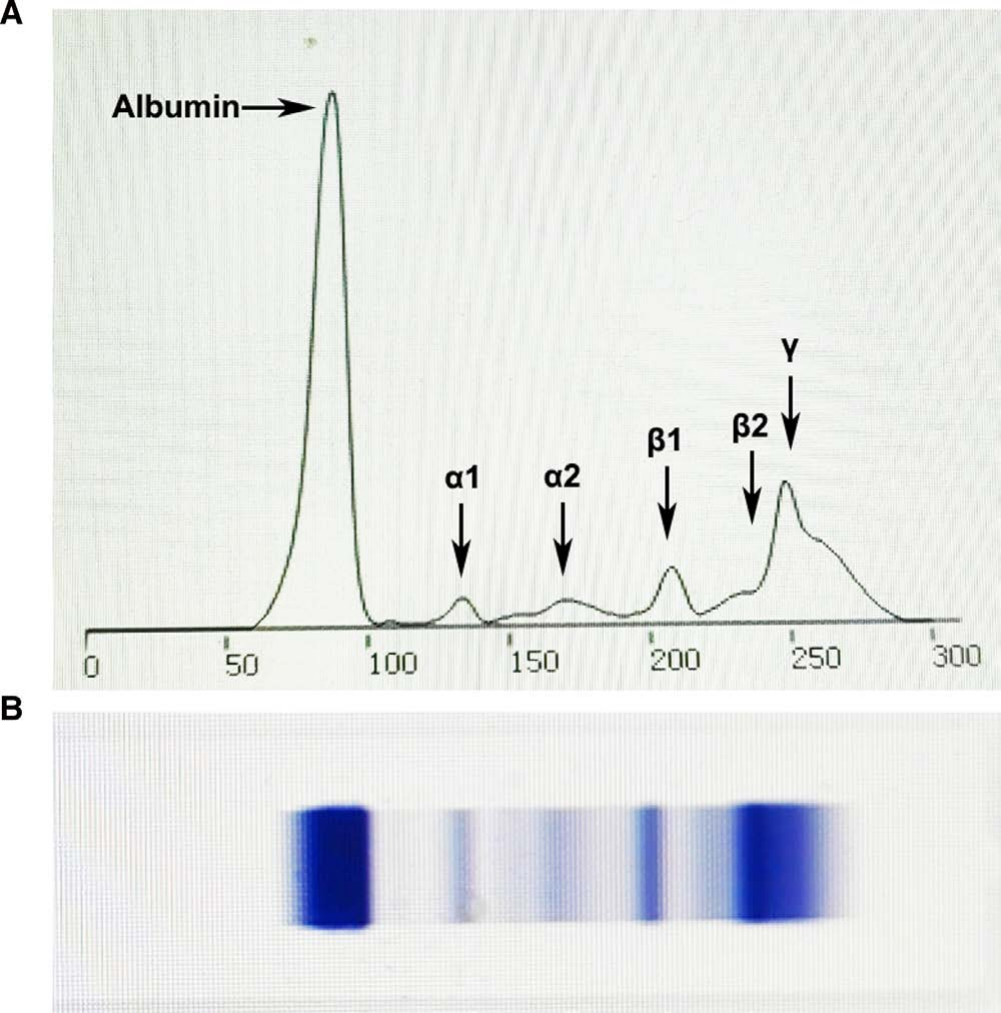
Capillary serum protein electrophoresis in the patient. (A) The arrow showed that γ had an abnormal monoclonal increase peak. (B) Electrophoresis bands of capillary serum protein electrophoresis.

Based on similar past experiences, it was inferred that the patient’s blood was likely to have high immunoglobulin IgM levels; therefore, we immediately contacted the patient and the cardiologist, hoping to perform additional tests, that is, serum protein electrophoresis and immunoglobulin analysis. As expected, the patient’s complement C3, C4, immunoglobulin G, and immunoglobulin A were normal, but IgM was significantly increased to 13,600 mg/L (reference range 400.00–2800.00 mg/L), and capillary serum protein electrophoresis showed that γ had an abnormal monoclonal increase peak (Table 2 and Fig. 1). Therefore, the patient was referred to the hematology department, where a lymph node color Doppler ultrasound was conducted. Blood light chain determination, blood immunization fixed electrophoresis, and urine immunofixation electrophoresis detection were also performed. Urine immunofixation electrophoresis was unremarkable, and blood immunofixation electrophoresis showed that LAMBDA had aggregation bands and an increased M protein percentage. The blood light chain assay showed that the light chain LAMBDA quantification was remarkably increased to 835 mg/dL (reference range 280–665.0 mg/dL) and that the light chain KAPPA was normal (Table 3). Therefore, the initial diagnosis was LPL/WM. Doctors suggested improving the pathological examination of enlarged lymph nodes, bone marrow examination, flow cytometry (lymphoma), FISH (MM + IGH), *MYD88*, and *CXCR4* fusion gene detection. FISH indicated that the patient was negative for *t* (14;16) (q32; q23), *t* (4;14) (q16; q32), and *t* (11;14) (q13; q32) as well as *1q21/RB1* and *D13S319/P53* genes. Flow cytometry results suggested that 2.5% of nucleated cells were likely abnormal phenotype clonal B cells. Forty-six chromosomes had no abnormalities, and the *MYD88* gene was positive. Repeated immunofixation electrophoresis showed IgM-LAMBDA positivity. Thus, the diagnosis of LPL/WM*^[8–11]^ was confirmed.

**Table 2:** Laboratory results part 2.

Parameter	Results	Reference interval	Units
Immunoglobulin G	10.10	7.00–16.00	g/L
Immunoglobulin A	1290.00	700.00–5000.00	mg/L
Immunoglobulin M	13600.00	400.00–2800.00	mg/L
Complement C3	955.00	790.00–1520.00	mg/L
Complement C4	321.00	100.00–400.00	mg/L
Serum protein electrophoresis			
Albumin	59.60	60.00–71.00	%
Alpha 1 globulin	2.50	1.40–2.90	%
Alpha 2 globulin	5.50	7.00–11.00	%
Beta 1 globulin	5.40	4.70–7.20	%
Beta 2 globulin	2.10	3.20–6.50	%
Gamma globulin	24.90	9.00–16.00	%

**Table 3:** Laboratory results part 3.

Parameter	Results	Reference interval	Units
Serum immunofixation electrophoresis			
Immunoglobulin A type κ M protein	Negative	Negative	N/A
Immunoglobulin A type λ M protein	Negative	Negative	N/A
Immunoglobulin G type κ M protein	Negative	Negative	N/A
Immunoglobulin G type λ M protein	Negative	Negative	N/A
Immunoglobulin M type κ M protein	Negative	Negative	N/A
Immunoglobulin M type λ M protein	Negative	Negative	N/A
Heavy chain M protein	Negative	Negative	N/A
Other	LAMBDA has a gathering band	Negative	N/A
Serum light chain			
kappa light chains	1170.00	598.00–1329.00	mg/dL
lambda light chain	835.00	280.00–665.00	mg/dL
Urine immunofixation electrophoresis			
Immunoglobulin A type κ M protein	Negative	Negative	N/A
Immunoglobulin A type λ M protein	Negative	Negative	N/A
Immunoglobulin G type κ M protein	Negative	Negative	N/A
Immunoglobulin G type λ M protein	Negative	Negative	N/A
Immunoglobulin M type κ M protein	Negative	Negative	N/A
Immunoglobulin M type λ M protein	Negative	Negative	N/A
Heavy chain M protein	Negative	Negative	N/A
Other	Negative	Negative	N/A

*LPL/WM is a rare, indolent mature B-cell lymphoma that accounts for <2% of non-Hodgkin’s lymphomas. LPL/WM is a lymphoma composed of small B lymphocytes, plasmacytoid lymphocytes, and plasma cells that often invade the bone marrow, lymph nodes, and spleen, and does not fit the diagnosis of other small B-cell lymphomas that might be associated with plasmacytic differentiation standards. WM is diagnosed when the LPL invades the bone marrow and is accompanied by serum monoclonal IgM gamma-globulin. Approximately 90% to 95% of LPL patients have WM, and only a small proportion of LPL patients secrete monoclonal immunoglobulin A, immunoglobulin G components, or secrete no monoclonal immunoglobulin at all.

## 3. Discussion

The serum LIH index, also known as the photometric test, was used for the semi-quantitative assessment of lipemia, icterus, and hemolysis in human serum or plasma using automatic biochemical analyzers. Patient samples were diluted with the LIH reagent (normal saline), and absorbance was measured at 6 wavelengths (450, 505, 570, 600, 630, and 700 nm). If one or more chromogens in a potentially interfering concentration were present in a sample, applicable flags were generated and reported along with the results of the analyses performed on that sample. These flags characterized the type of chromatic substance (LIP: lipemia/turbidity; ICT: bilirubin/icterus; HEM: hemoglobin) and the approximate concentration of the interferent (e.g., +, ++, ++++). The serum LIH index was used to measure the degree of chyle, hemolysis, and jaundice in serum samples, and to obtain the indices L, H, and I. Absorbance values represented by different LIH degrees are shown in Supplementary material (Table S1, Supplemental Digital Content, http://links.lww.com/MD/O220).

When the patient first saw a doctor for dizziness, because of his old age he chose the Department of Cardiology and visited a specialist outpatient clinic. The initial tests were also routine tests, and the results were not abnormal. If dizziness caused by the common carotid artery or cervical spine is treated, its effect may not be obvious. Waiting for the final diagnosis of obvious blood abnormalities or other abnormalities, the patient may miss the best time for diagnosis and treatment, or waste money and waiting time waiting for diagnosis and treatment.

We deduced that M protein made the lipemia positive because IgM mostly exists in the form of pentamers, and M protein in this form is more likely to form turbidity in the monitoring system and interfere with detection.^[12]^ In this case, there was in fact a high level of IgM, high enough to cause turbidity. The reagent for assaying serum LIH index was normal saline, which was diluted with deionized water and mixed with the sample. The deionized water contained almost no ionic impurities, which changed the original ionic strength and pH value of the sample, causing M protein to separate out in the system. This produced turbidity and lead to an increase in the blank absorbance reading, resulting in positive lipemia.

At present, the automatic serum LIH index is widely used in biochemical detection. Through the detection, the samples characteristics can be observed, to a certain extent, in the assembly line. Special attention must be drawn to the discrepancy between serum LIH index and sample character, especially regarding the lipemia index. The difference between the lipemia index and the sample usually occurs within the following 2 cases: the first, similar to the case report, can be seen in patients with M-proteinemia with high lipemia index, normal triglycerides and sample appearance^[13]^; the second, when using the enzyme method to detect triglycerides other glycerides (such as double triglycerides, single triglycerides or free glycerol) might be present, resulting in a detection falsely increased, therefore, the serum lipemia index will be shown as normal but with high triglycerides.^[14]^ These inconsistent specimens can be found on time to optimize the patient’s diagnosis and treatment.

In practice, we’ve identified patients with various undiagnosed conditions, including persistent skin itching, suspected coronary heart disease, asymptomatic cases during health checks, and misdiagnosed gout. Through timely communication, we directed them to hematology for accurate diagnosis, uncovering root causes even when initial tests seemed normal.

In addition, because of its unique physical and chemical properties, M protein can precipitate under specific conditions, which could interfere with colorimetric and turbidity analysis and affect the results of in vitro detection. In recent years, many studies have reported that M protein interferes with biochemical detection, including C-reactive protein and uric acid.^[13,15,16]^ If such interference cannot be timely detected, it will lead to a deviation in the results. Therefore, the development of a serum LIH index, especially in the application of lipemia index, would bring great benefits.

The limitation of this case report was that the patient did not return to the hospital for follow-up treatment, therefore, there was no further observation of the effect of M protein on serum LIH index after treatment.

## 4. Conclusion

We are the first to report and summarize the role of serum index, particularly lipemia index, in clinical diagnosis and treatment. This case report shows that M protein recognition could result in an abnormal serum LIH index while other parameters remain normal. The serum LIH index is not only a sample property-monitoring indicator but it can also, to a certain extent, detect subtle early disease changes and eliminate interference. More importantly, it can provide ideas to optimize patient diagnosis.

## Acknowledgments

We would like to thank Jingzhong Liao and Wenli Li for their guidance on this case report.

## Author contributions

**Conceptualization:** Bin Yi.

**Data curation:** Kun Wang.

**Supervision:** Bin Yi.

**Writing – original draft:** Kun Wang.

**Writing – review & editing:** Bin Yi.

## Supplementary Material


