# Supplementary material for: Uncovering a lymphoplasmacytic lymphoma/Waldenström macroglobulinemia initially manifesting as dizziness detected through abnormal serum lipemia index: A case report
Source: Medicine (Baltimore). 2024 Dec 20;103(51):e40999. doi: 10.1097/MD.0000000000040999 (PMC11666197; doi:10.1097/MD.0000000000040999)
Supplement: Supplementary file 1 [file medi-103-e40999-s001.docx]

**Table S1. Absorbance values represented by different degrees of LIH**

| **LIH Judgement Level** | **Lipemia** | **Icterus** | **Hemolysis** |
| --- | --- | --- | --- |
| **+** | 0.0150 | 0.1100 | 0.1700 |
| **++** | 0.0400 | 0.2100 | 0.3500 |
| **+++** | 0.0800 | 0.4300 | 0.7000 |
| **++++** | 0.1200 | 0.8500 | 1.0000 |
| **+++++** | 0.2000 | 1.7000 | 1.7000 |
